# Pressure-induced anomalous valence crossover in cubic YbCu_5_-based compounds

**DOI:** 10.1038/s41598-017-06190-3

**Published:** 2017-07-19

**Authors:** Hitoshi Yamaoka, Naohito Tsujii, Michi-To Suzuki, Yoshiya Yamamoto, Ignace Jarrige, Hitoshi Sato, Jung-Fu Lin, Takeshi Mito, Jun’ichiro Mizuki, Hiroya Sakurai, Osamu Sakai, Nozomu Hiraoka, Hirofumi Ishii, Ku-Ding Tsuei, Mauro Giovannini, Ernst Bauer

**Affiliations:** 1 RIKEN SPring-8 Center, RIKEN, 1-1-1 Kouto, Sayo, Hyogo 679–5148 Japan; 2 International Center for Materials Nanoarchitectonics (MANA), National Institute for Materials Science, 1-2-1 Sengen, Tsukuba, Ibaraki 305–0047 Japan; 3 RIKEN Center for Emergent Matter Science, RIKEN, 2-1, Hirosawa, Wako, Saitama 351–0198 Japan; 4 Graduate School of Science and Technology, Kwansei Gakuin University, Sanda, Hyogo 669–1337 Japan; 5 Photon Sciences Directorate, Brookhaven National Laboratory, Upton, New York 11973 USA; 6 HiSOR, Hiroshima University, Kagamiyama 2-313, Higashi-Hiroshima, 739–8526 Japan; 7 Department of Geological Sciences, The University of Texas at Austin, Austin, Texas 78712 USA; 8 Center for High Pressure Science and Technology Advanced Research (HPSTAR), Shanghai, 201203 China; 9 Graduate School of Material Science, University of Hyogo, Sayo, Hyogo 678–1297 Japan; 10 National Institute for Materials Science, 1-2-1 Sengen, Tsukuba, 305–0047 Japan; 11 National Synchrotron Radiation Research Center, Hsinchu, 30076 Taiwan; 12 CNR-SPIN, Dipartimento di Chimica e Chimica Industriale, University of Genova, Genova, Italy; 13 Institute of Solid State Physics, Vienna University of Technology, 1040 Wien, Austria

## Abstract

A pressure-induced anomalous valence crossover without structural phase transition is observed in archetypal cubic YbCu_5_ based heavy Fermion systems. The Yb valence is found to decrease with increasing pressure, indicating a pressure-induced crossover from a localized 4*f* ^13^ state to the valence fluctuation regime, which is not expected for Yb systems with conventional *c*–*f* hybridization. This result further highlights the remarkable singularity of the valence behavior in compressed YbCu_5_-based compounds. The intermetallics Yb_2_Pd_2_Sn, which shows two quantum critical points (QCP) under pressure and has been proposed as a potential candidate for a reentrant Yb^2+^ state at high pressure, was also studied for comparison. In this compound, the Yb valence monotonically increases with pressure, disproving a scenario of a reentrant non-magnetic Yb^2+^ state at the second QCP.

## Introduction

**Figure 1:**
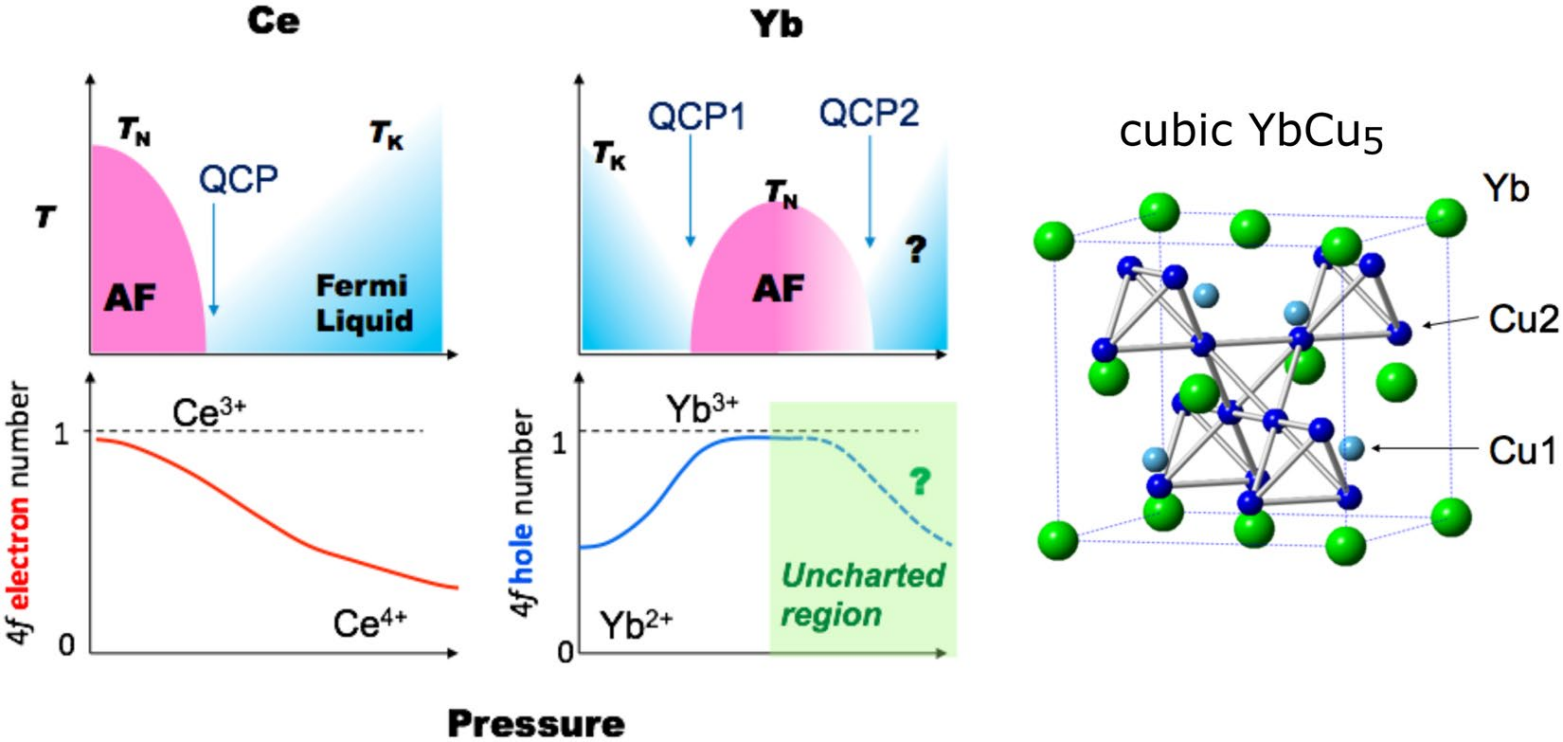
Schematic of the pressure-temperature phase diagrams and pressure dependence of 4*f* electron numbers for Yb and Ce systems, where *T*_N_, *T*_K_, and AF are Néel temperature, Kondo temperature, and antiferromagnetic ordered sate, respectively^3, 5^. In the Yb system, two quantum critical points (QCPs) are possibly observed. An image of the crystal structure of cubic YbCu_5_ is also shown.

Material properties strongly correlate to the spin, orbital, and charge degrees of freedom of the electrons. In intermetallic rare-earth compounds, valence fluctuations provide an additional degree of freedom to pressure or temperature-driven ground states. Physical properties in the valence fluctuation systems can be understood in terms of a competition between the Ruderman-Kittel-Kasuya-Yosida (RKKY) interaction and the Kondo effect, both are originated by interaction between *f* and conduction (*c*) electrons^1, 2^. Pressure is a powerful and clean tool to directly tune the Kondo temperature or *c*–*f* hybridization strength. In Yb compounds, commonly the magnetic Yb^3+^ state is favored at high pressures due to its smaller ionic radius compared with Yb^2+^. Interestingly, the rare-earth metal theory predicts a return to the divalent state or to the valence fluctuation region with further increase of the pressure up to a few hundreds GPa (Mbar range)^3^, which has not been observed experimentally yet, despite trials up to 202 GPa in Yb metal^4^. Figure 1 shows schematic of the pressure-temperature phase diagrams and pressure dependence of 4*f* electron numbers for Yb and Ce systems, together with a sketch of the crystal structure of cubic YbCu_5_^5^. In Ce systems, pressure induces a non-magnetic ground state, while in Yb systems, a return to the Yb^2+^ state at high pressures would be consistent with the increase of Kondo temperature (*T*_K_).

YbCu_5_ -based intermetallic compounds are well known archetypal *f*-electron heavy-fermion systems. A wide diversity of interesting physical phenomena was reported upon Cu-site substitution in the cubic Yb*M*Cu_4_ systems (*M* = In, Ag, Au, etc.)^6, 7^, such as a temperature-induced first-order valence transition in YbInCu_4_, Kondo lattice effects in YbAgCu_4_, and antiferromagnetic order in YbAuCu_4_^8–10^. In the mother material cubic YbCu_5_, the low-temperature physical properties have been described employing a Kondo lattice with a heavy Fermi liquid ground state^11–13^. The rich variety of physical properties seems to stem primarily from the intermediate valent ground state of Yb^6^. However, pressure-induced changes in the electronic structure of YbCu_5_ are still unexplored. It is notably that the Yb valence distinctly depends on its crystal structure: cubic and hexagonal YbCu_5_ exhibit Yb valences of nearly 3+ (ref. 11) and ~2.5 (ref. 14), respectively. On the other hand, as a novel ternary Yb heavy-fermion compound, Yb_2_Pd_2_Sn is known to exhibit two pressure-driven quantum critical points (QCPs)^15, 16^. A scenario based on the single impurity Anderson model (SIAM) taking into account a pressure-induced enhancement of valence fluctuations at low pressure and suppression at high pressure was suggested to explain the two QCPs^17^. Yb_2_Pd_2_Sn possesses a tetragonal crystal structure with two types of layers that alternatively stack along *c*-axis. Another scenario based on the geometrical frustration forming the Shastry-Sutherland lattice^18^ has been proposed, beyond the normal framework of competition between the RKKY interaction and the Kondo effect^19^. Still, the precise origin of the two QCPs in Yb_2_Pd_2_Sn is not fully elucidated.

In this paper we report a comparative study of the pressure-induced valence crossover in cubic YbAg_*x*_Cu_5−*x*_ (*x* = 0, 0.5, and 1.0) and Yb_2_Pd_2_Sn. Electron probe microanalyses showed chemical compositions according to Yb_0.98_Cu_5.02_, Yb_0.984_Ag_0.504_Cu_4.51_, and Yb_0.99_Ag_0.93_Cu_4.08_. External pressure is advantageous in that the Kondo temperature can be controlled uniformly, whereas chemical pressure can easily induce local distortions. We employ x-ray absorption spectroscopy in the partial fluorescence yield mode (PFY-XAS) and resonant x-ray emission spectroscopy (RXES) to derive the Yb valence as a function of pressure^20^. The results are combined with x-ray diffraction (XRD) measurements. We find an anomalous pressure-induced decrease of the valence in cubic YbCu_5_-based compounds followed by a valence increase at higher pressures, without structural phase transition. In Yb_2_Pd_2_Sn, the Yb valence increases monotonically with pressure at low temperature, disproving a return to the Yb^2+^ state at the second QCP.

## Results and Discussion

**Figure 2:**
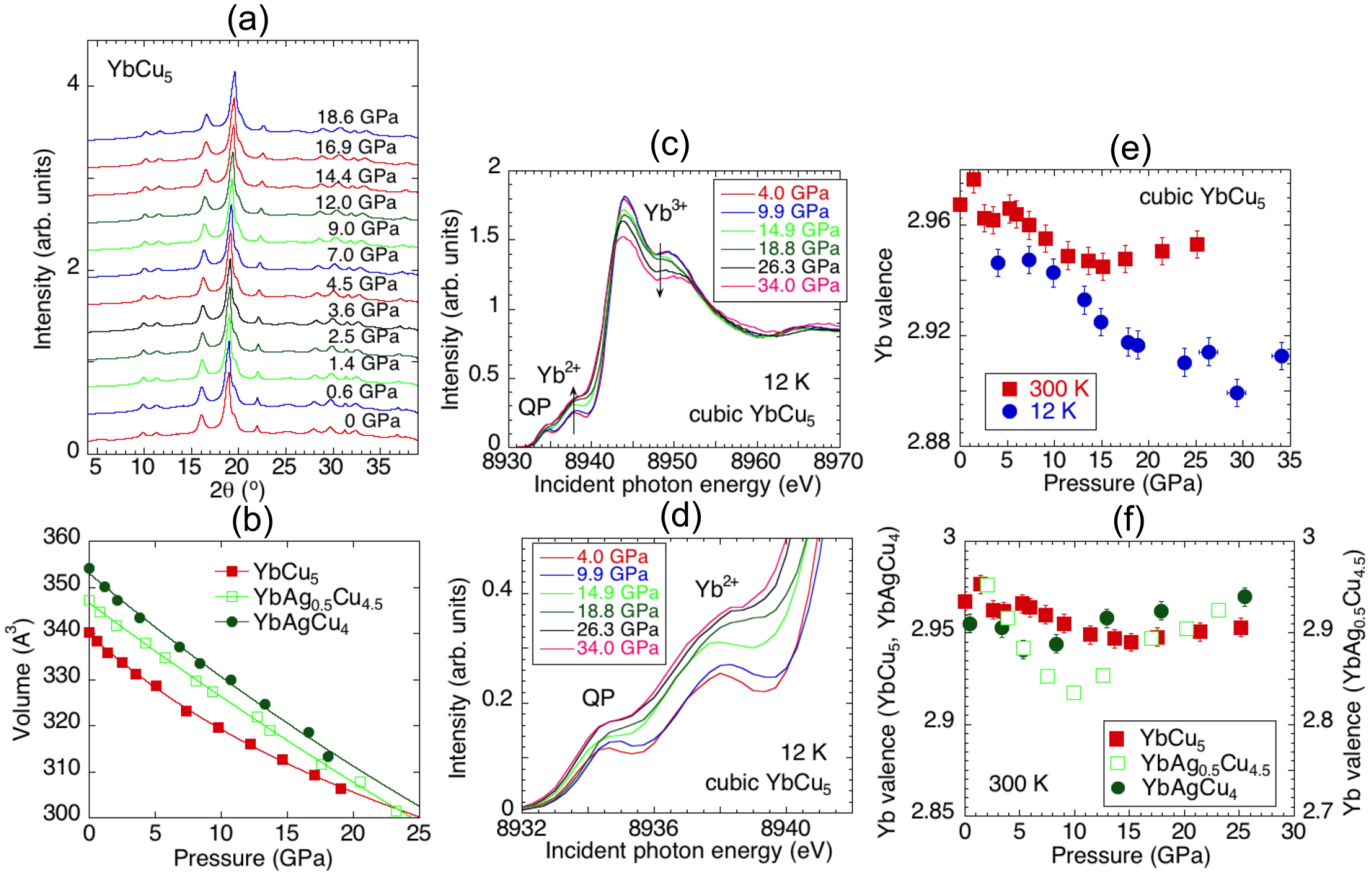
The experimental results of cubic YbAg_*x*_Cu_5−*x*_ (*x* *=* 0, 0.5, and 1.0) at 300 K are shown. (**a**) X-ray diffraction patterns measured with *λ* = 0.6888 Å for YbCu_5_. (**b**) Pressure dependence of the volume for YbAg_*x*_Cu_5−*x*_ (*x* = 0, 0.5, and 1.0). Solid lines are fits with the equation of state. (**c**) Pressure dependence of PFY-XAS spectra at 12 K for YbCu_5_. (**d**) Enlarged view of (**c**) for the quadrupole (QP) and Yb^2+^ components. (**e**) Yb valence estimated from the fit to the PFY-XAS spectra for cubic YbCu_5_ at 300 and 12 K. (**f**) Pressure dependence of the Yb valence for YbAg_*x*_Cu_5−*x*_ (*x* = 0, 0.5, and 1.0) at 300 K.

Figure 2 shows the XRD and PFY-XAS measurements for cubic YbAg_*x*_Cu_5−*x*_ (*x* = 0, 0.5, and 1.0). The XRD patterns in Fig. 2(a) evidence a cubic crystal structure of YbCu_5_; no pressure-induced structural transitions were observed for the three YbCu_5_-based compounds in the pressure range measured. The volume of the three compounds monotonically decreases with pressure as shown in Fig. 2(b). This behavior is consistent with previous reports^21^. Figure 2(c) and (d) show the pressure dependence of the PFY-XAS at 12 K for YbCu_5_. The pressure dependence of the mean Yb valence derived from the fits of the PFY-XAS spectra is shown in Fig. 2(e) and (f). In YbCu_5_ the Yb valence at 300 K decreases when the pressure is increased up to around 10–15 GPa, and show an increasing trend with further increase of the pressure, although the change in the valence is within the experimental errors. This increasing trend of the Yb valence at high pressures is observed clearly in YbAg_0.5_Cu_4.5_ above 10 GPa. Valence fluctuations in YbCu_5_ become enhanced at 12 K, keeping the same trend as that at 300 K as shown in Fig. 2(f). In YbAg_0.5_Cu_4.5_ and YbAgCu_4_ similar pressure-induced changes in the Yb valence are observed and the pressure dependent minima of the Yb valences occur around 10 and 5 GPa, respectively. The RXES spectra were measured at *hv* = 8938 eV, which corresponds to the Yb^2+^ resonance incident photon energy, where the the intensity of Yb^2+^ is highest (see supplementary information). The intensity ratio between Yb^3+^ and Yb^2+^ in the RXES spectra closely follows the trend consistent with the Yb valence as a function of pressure. This isostructural valence change, which has never been reported in the literature for any other valence fluctuating compound, is highly anomalous, since the smaller-radius Yb^3+^ ion is expected to be favored under high pressure. We note that a pressure-induced reentrant transition to a lower valence state had been previously reported in EuO, albeit accompanied by a structural transition^22^. This transition, well described by first-principle band calculations^23^, is therefore different in nature compared to the isostructural transition in YbCu_5_.

In cubic YbCu_5_ the electrical resistivity at ambient pressure follows the Fermi liquid power law *ρ*(*T*) = *ρ*_0_ + *AT* ^2^ below the temperature *T*_FL_ ≈ 40 K^13^; *ρ*_0_ is the residual resistivity and *A* is the quadratic term coefficient. The pressure dependence of *A* below 4 GPa was reported to show a divergent behavior with pressure; above 5 GPa a non-Fermi liquid state was predicted^13^. This suggests that there might be a QCP in cubic YbCu_5_ around 5–6 GPa. In YbCu_2_Si_2_ (ref. 24) and YbNiGe_3_ (ref. 25), the Yb valence increases with pressure and shows a pronounced change in the slope around the QCP. Our results in YbCu_5_ show that the valence stabilizes around 5 GPa and decreases slightly at 5–15 GPa. The resistivity was measured up to 4 GPa^13^ and measurements at higher pressures confirm the presence of a QCP.

The calculated effective magnetic moment of cubic YbCu_5_, assuming a total angular momentum *j* = 7/2, is 4.53 *μ*_B_. Curie-Weiss fit to the magnetic susceptibility of cubic YbCu_5_ for *T* > 150 K reveals a Weiss temperature of -26 K and an effective magnetic moment of 4.43 *μ*_B_^11^. This indicates a nearly trivalent Yb state and supporting the above results at ambient pressure. The increase of the Yb valence above 5–15 GPa in Fig. 2(f) seems to demonstrate a return to the region where the Yb^3+^ state is stable as shown in Fig. 1. In Yb compounds, the SIAM or the Anderson lattice model (ALM) has successfully explained various phenomena related to the *c*–*f* interaction for now several decades. In our previous study of the temperature dependence of the Yb valence in cubic YbCu_5_ at ambient pressure, the experimentally-derived valences were compared with estimations based on the SIAM^14^. The SIAM was found to reproduce satisfactorily the temperature dependent Yb valence. However, our high-pressure study of cubic YbCu_5_ cannot be understood with a simple scenario based on the Anderson model.

Recently, anomalous temperature dependences of the Yb valence have been also reported for the Yb compounds like Yb_*x*_Fe_4_Sb_12_^20^ and YbMn_6_Ge_6−*x*_Sn_*x*_^26^. For the latter case, a scenario based on the presence of magnetically ordered Mn moments and on an Anderson Hamiltonian with a Zeeman term modeling the magnetic interactions was proposed to explain the unusual temperature dependence^26^. Note that in cubic YbCu_5_ such magnetically ordered moments do not exist.

We performed density functional theory (DFT) calculations at 0, 10, and 20 GPa for cubic YbCu_5_. Details are summarized in the supplementary information. Increasing pressure results in a broadening of the conduction band and of the Yb 4*f* states around the Fermi level through hybridization typically in the orbital density of sates (DOS) at the Fermi level of Cu2. The electron numbers in the Muffin tin sphere decrease with pressure, which, however, does not explain the present results. While the change in the DOS under pressure reduces the *f* electron number within the approximation as commonly expected, the broadening of the band width together with the *c*–*f* hybridization can cause a stabilization of the nonmagnetic *f* ^14^ states as discussed below. Calculations using the large degeneracy expansion method suggested that the characteristic temperature related to the Kondo effect, *T*_0_, can be expressed as^27^:

1 $${T}_{0}=D{g}^{\mathrm{1/6}}{e}^{-\mathrm{1/6}g}{(D/{\rm{\Delta }})}^{\mathrm{8/6}},$$

where *D*, Δ are the width of the conduction band and the energy of the spin-orbit coupling, respectively. Also, *g* = Γ/*π*|*ε*_*f*_|, where Γ is the hybridization strength for the *f* and conduction electrons, and *ε*_*f*_ is the energy of the *f* level. This relation is valid for degeneracy *N* = 6 systems like Ce^3+^ Kondo lattices. Here, we assumed that the spin-orbit coupling Δ is much larger than *T*_0_. In this relation, the characteristic temperature *T*_0_ can increase through the bandwidth *D* and the hybridization strength Γ. Although the actual change of the Kondo temperature with pressure can be more complex, this pressure-induced enhancement of *T*_0_, which stabilizes the nonmagnetic *f* ^14^ state, may be one possible explanation for the decrease of the Yb valence in YbCu_5_-based compounds under pressure. First-principles calculations considering the local dynamical correlation may reproduce the situation, and a study based on DFT + dynamical mean field theory considering the strong spin-orbit coupling effect with the accurate impurity solver is a future task.

We emphasize that the interplay of the *f* states with peculiar features of the band structure near the Fermi level can cause a variety of intriguing phenomena beyond the understanding of the conventional *c*–*f* hybridization framework. For example, the anomalous valence transition in Yb_*x*_Fe_4_Sb_12_ and YbMn_6_Ge_6_ were not understood by a normal Kondo-lattice picture. Instead, it was necessary taking into account the effect of distinct band structure features or of magnetism related to *d* electrons. In pure rare-earth metals, the re-entrance pressure to Yb^2+^ state is extremely high, but in rare-earth compounds this value is possibly reduced to lower pressures. Here, we stress that the Yb valence started to decrease already at much lower pressure. Thus, the anomalous valence change induced by pressure in YbCu_5_-based compounds also calls for more detailed experimental and theoretical studies.

**Figure 3:**
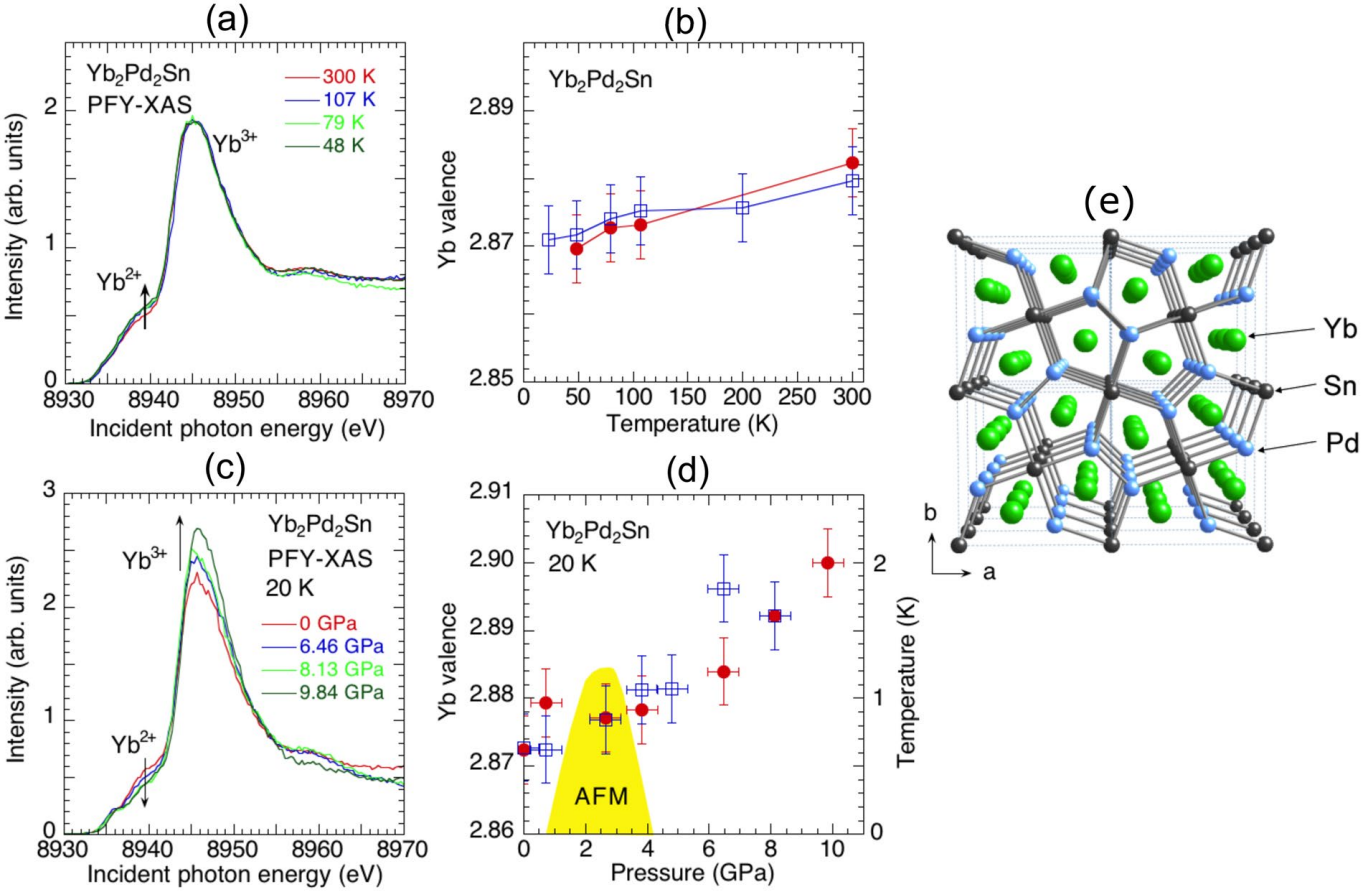
The experimental results of Yb_2_Pd_2_Sn are shown. (**a**) Temperature dependence of the PFY-XAS spectra at ambient pressure. Arrows in (**a**) correspond to the direction to decrease the temperature. (**b**) Temperature dependence of the Yb valence estimated from the fits to the PFY-XAS spectra (closed circle) and the RXES spectra (open square) at *hv* = 8938 eV. (**c**) Pressure dependence of the PFY-XAS spectra at 20 K. (**d**) Pressure dependence of the Yb valence estimated from the fits to the PFY-XAS spectra (closed circle) and the RXES spectra (open square). In (**d**) we also show the pressure dependence of the Néel temperature as a yellow-colored area, where the data are taken from the literature^16^. (**e**) Crystal structure of Yb_2_Pd_2_Sn.

Figure 3 shows various results of Yb_2_Pd_2_Sn at temperatures from 20 to 300 K. Due to technical limitations of the membrane-driven DAC in our cryostat, the lowest temperature reached in this study is is well above 0 K, where the QCP-related behavior dominates. The Yb valence decreases slightly with temperature down to 23 K as shown in Fig. 3(a) and (b)^28^; the hybridization, however, is stronger at low temperatures. This decrease is expected to be prolonged also below 20 K as the hybridization strength just slightly increases. Based on the pressure dependence of the PFY-XAS spectra in Fig. 3(c) and RXES spectra (not shown here), the Yb valence is found to monotonically increase with pressure as shown in Fig. 3(d). Pressure thus suppresses the valence fluctuations of the Yb ions, driving them towards a Yb^3+^ state, which results in a decrease of the Kondo temperature. The two-QCP scenario suggests that the *c*–*f* hybridization is enhanced beyond the second QCP at high pressure, resulting in an increase of *T*_*K*_^17^, and hinting here at a return into a valence fluctuation region. Our results actually deny this possibility, leaving the geometrical frustration as a more plausible scenario for the origin of the two QCPs of Yb_2_Pd_2_Sn. This result further stresses the uniqueness of the valence behavior in cubic YbCu_5_-based compounds under pressure.

In conclusion, a highly anomalous isostructural pressure-induced decrease of the valence was observed in YbCu_5_-based compounds. The result cannot be explained within the framework of the common *c*–*f* hybridization mechanism. In contrast, the pressure dependence of the Yb valence in Yb_2_Pd_2_Sn shows a smooth increase of the Yb valence with pressure which makes a reentrant valence fluctuation scenario unlikely to explain the second QCP. Low-temperature data for the Ag-substituted systems may be helpful to understand the pressure-induced anomalous valence transition of the Yb systems and the Kondo physics under pressure.

## Methods

Cubic YbCu_5_ sample was prepared by argon arc melting and subsequent annealing at 850°C for 2 hours under high pressure of 6 GPa^11^. The chemical composition of YbCu_5_ was Yb_0.98_Cu_5.02_ as determined by electron probe microanalysis (EPMA). Polycrystalline samples of YbAgCu_4_ and YbAg_0.5_Cu_4.5_ were prepared by melting in an argon arc furnace and subsequent annealing at 800 °C in evacuated silica tubes. Polycrystalline samples of Yb_2_Pd_2_Sn were prepared in a closed tantalum-tube with Ar atmosphere at 1300 °C for 1.5 hours by a high-frequency induction furnace and then annealed at 980 °C for 10 days.

The pressure dependence of the x-ray diffraction patterns were measured at BL12B1, SPring-8, using a 3-pin plate diamond anvil cell (DAC, Almax Industries) with a CCD detection system at room temperature. We applied an arrangement of both incoming and outgoing x-ray beams passing through the diamonds with an incident photon energy of *hv* = 18 keV (*λ* = 0.6888 Å). A two dimensional image of the CCD system was integrated by using the FIT2D program^29^. The diffraction patterns were analyzed by the Rietveld method using the RIETAN-FP program^30, 31^.

PFY-XAS and RXES measurements were performed at the Taiwan beamline BL12XU, SPring-8. Details of the experimental setup have been published elsewhere^32^. The overall energy resolution was estimated to be about 1 eV around the emitted photon energy of 7400 eV from the elastic scattering. A closed-circuit He cryostat was used for the low-temperature measurements down to 20 K. The high-pressure conditions were realized using a diamond anvil cell (DAC) with a Be-gasket; the pressure-transmitting medium was silicone oil. A membrane-controlled DAC was used for high pressure experiments at low temperatures. The pressure was measured based on the Raman shift of the ruby fluorescence.

The Yb mean valence is estimated by integrating the area of each charge state of the PFY-XAS spectra. The mean valence is defined to be *v* = 2 + *I*(3+)/(*I*(2+) + *I*(3+)), where *I*(n+) is the intensity of Yb^n+^ component. An example of such evaluations is shown in the supplementary information. The error of the valence mainly comes from the statistics of the total counts and fit errors, which was of the order of less than 0.2–0.5%.

The electronic structure calculations are implemented in the WIEN2k program code with the all-electron full-potential linear augmented plane wave method using the exchange-correlation functional proposed by Perdew, Burke, and Ernzerhof ^33^ for the cubic YbCu_5_ under the pressure at 0, 10, and 20 GPa. Detailed results are shown in the supplementary information.

## Electronic supplementary material

Supplementary information
