# Supplementary material for: Pressure-induced anomalous valence crossover in cubic YbCu5-based compounds
Source: Sci Rep. 2017 Jul 19;7:5846. doi: 10.1038/s41598-017-06190-3 (PMC5517414; doi:10.1038/s41598-017-06190-3)
Supplement: Supplementary file 1 — Supplementary information [file 41598_2017_6190_MOESM1_ESM.pdf]

Supplementary information: Pressure-induced anomalous valence crossover in cubic $YbCu_5$-based compounds

Hitoshi Yamaoka,[1] Naohito Tsujii,[2] Michi-To Suzuki,[3] Yoshiya Yamamoto,[4] Ignace Jarrige,[5] Hitoshi Sato,[6] Jung-Fu Lin,[7,8] Takeshi Mito,[9] Jun'ichiro Mizuki,[4] Hiroya Sakurai,[10] Osamu Sakai,[11] Nozomu Hiraoka,[12] Hirofumi Ishii,[12] Ku-Ding Tsuei,[12] Mauro Giovannini,[13] and Ernst Bauer[14]

[1] *RIKEN SPring-8 Center, RIKEN, 1-1-1- Kouto, Sayo, Hyogo 679-5148, Japan*
[2] *International Center for Materials Nanoarchitectonics (MANA), National Institute for Materials Science, 1-2-1 Sengen, Tsukuba,Ibaraki 305-0047, Japan*
[3] *RIKEN Center for Emergent Matter Science, 2-1, Hirosawa, Wako, Saitama 351-0198, Japan*
[4] *Graduate School of Science and Technology, Kwansei Gakuin University, Sanda, Hyogo 669-1337, Japan*
[5] *Photon Sciences Directorate, Brookhaven National Laboratory, Upton, New York 11973, USA*
[6] *Hiroshima Synchrotron Radiation Center, Hiroshima University, Kagamiyama 2-313, Higashi-Hiroshima 739-8526, Japan*
[7] *Department of Geological Sciences, The University of Texas at Austin, Austin, Texas 78712, USA*
[8] *Center for High Pressure Science and Technology Advanced Research (HPSTAR), Shanghai 201203, China*
[9] *Graduate School of Material Science, University of Hyogo, Sayo, Hyogo 678-1297, Japan*
[10] *National Institute for Materials Science, 1-1 Namiki, Tsukuba, Ibaraki 305-0044, Japan*
[11] *National Institute for Materials Science, 1-2-1 Sengen, Tsukuba 305-0047, Japan*
[12] *National Synchrotron Radiation Research Center, Hsinchu 30076, Taiwan*
[13] *CNR-SPIN, Dipartimento di Chimica e Chimica Industriale, University of Genova, Italy*
[14] *Institute of Solid State Physics, Vienna University of Technology, 1040 Wien, Austria*

XRD PATTERNS AND PRESSURE DEPENDENCE OF THE VOLUME

We performed x-ray diffraction (XRD) for $YbAg_xCu_{5-x}$ (x = 0, 0.5, and 1.0) at 300 K under pressure. The XRD patterns of cubic $YbCu_5$ and hexagonal $YbCu_{6.5}$ are shown in Fig. 1, measured with a Cu $K\alpha$ radiation at 300 K. [1] The x-ray pattern of hexagonal $YbCu_5$ (upper panel, Fig. 1) are distinctly different from those of cubic $YbCu_5$ (lower panel, Fig. 1), confirming the differences of both crystal structures.

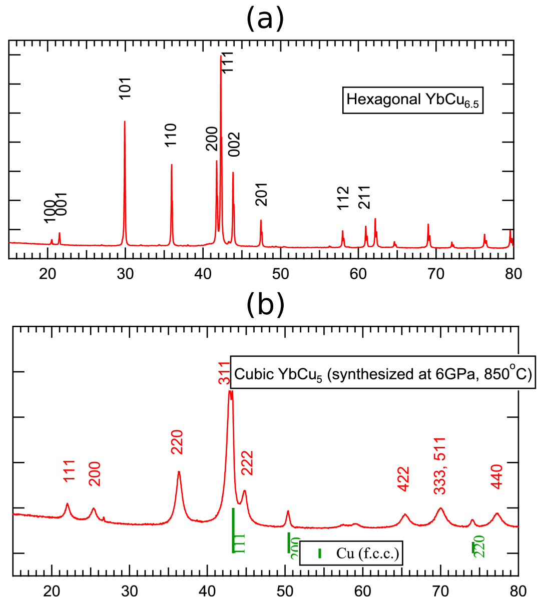

FIG. 1. (Color online). XRD diffraction patternsa of cubic $YbCu_5$ and hexagonal $YbCu_{6.5}$ measured with a Cu $K\alpha$ line at ambient pressure and 300 K.

Figure 2 shows the diffraction patterns for $YbAg_{0.5}Cu_{4.5}$ and $YbAgCu_4$ as a function of pressure. The diffraction peaks of $YbAg_{0.5}Cu_{4.5}$ and $YbAgCu_4$ are sharper than those of cubic $YbCu_5$. The line-broadening in cubic $YbCu_5$ may be induced by

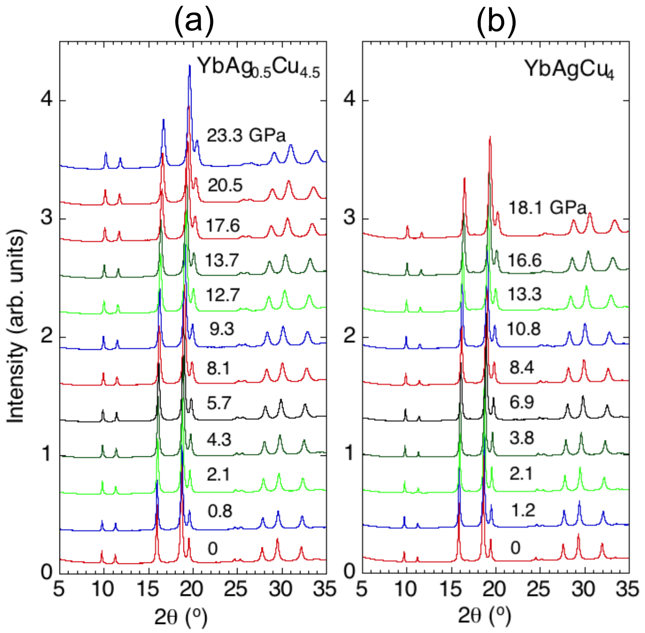

FIG. 2. (Color online). X-ray diffraction patterns measured with $\lambda = 0.6888$ Å for (a) $YbAg_{0.5}Cu_{4.5}$ and (b) $YbAgCu_4$.

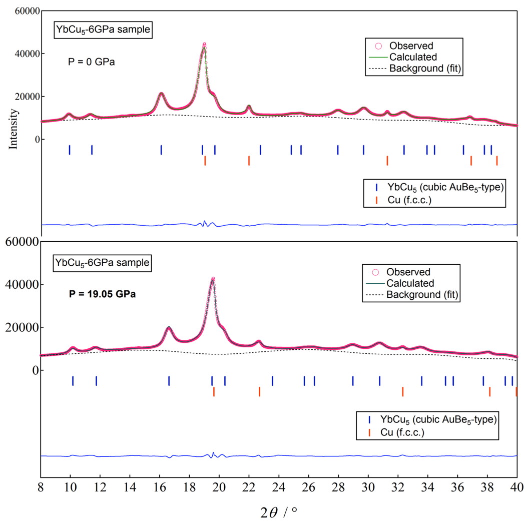

FIG. 3. (Color online). Examples of the XRD spectra of cubic $YbCu_5$ at 0 and 19.05 GPa (open circles) with the fits (solid lines). Difference between the experimental data and the calculated result is shown in lower part of each figure.

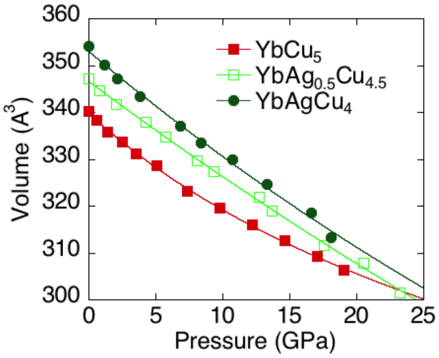

FIG. 4. (Color online). Relationship between the volume (V) and pressure of cubic $YbCu_5$, where V_0 is the volume at ambient pressure. The solid line is a fit of the equation of state to the experimental data.

the process making smaller-sized powder to avoid the hot spots in the two-dimensional diffraction patterns. However, the pattern are still sufficient to study whether or not there is a structural transition. Examples of the fits at 0 and 19.05 GPa for $YbCu_5$ are shown in Fig. 3. There are no structural transitions within the pressure range measured for $YbAg_xCu_{5-x}$ ($x = 0$, 0.5, and 1.0).

Figure 4 shows fits of the pressure-volume relation by using an empirical formula of the equation of state.

$$\frac{V}{V_0} = [1 + p\frac{B_0{}'}{B_0}]^{-\frac{1}{B_0{}'}} \quad (1)$$

where P, V, V_0, B_0, and $B_0{}'$ are pressure, volume, volume at ambient pressure, bulk modulus of incompressibility, and fits first derivative with respect to the pressure,

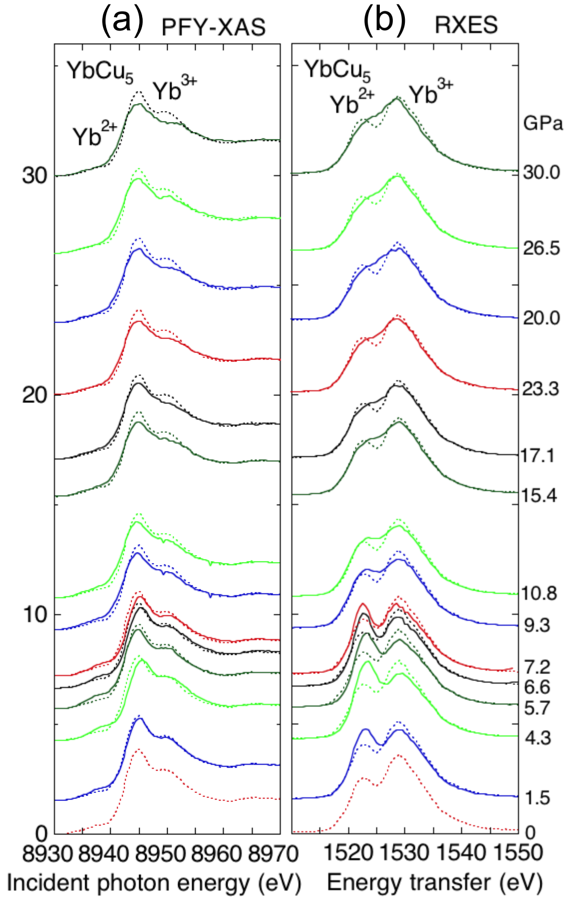

FIG. 5. (Color online). Pressure dependence of (a) the PFY-XAS spectra and (b) the RXES spectra of $YbCu_5$ at 300 K.

respectively. The following parameters are obtained: $B_0 = 127.1$ GPa, $B_0{}' = 6.91$, $V_0 = 339.9$ Å for cubic $YbCu_5$, $B_0 = 162.5$ GPa, $B_0{}' = 0.558$, $V_0 = 346.62$ Å for $YbAg_{0.5}Cu_{4.5}$, and $B_0 = 145.7$ GPa, $B_0{}' = 1.33$, $V_0 = 353.03$ Å for cubic $YbAgCu_4$.

PFY-XAS AND RXES SPECTRA

In Fig. 5 we show the pressure dependence of the PFY and RXES spectra of $YbCu_5$ at 300 K. The RXES spectra of $YbCu_5$ were measured around the incident energy of the peak of the Yb_{2+} component. The pressure-induced behavior of the each Yb component of the REXS spectra is very similar to that of the PFY-XAS spectra.

Figure 6 shows the pressure dependence of the PFY-XAS spectra of (a) $YbAg_{0.5}Cu_{4.5}$ and (b) $YbAgCu_4$ at 300 K. In Figs. 6(a) and 5(b) the dotted lines correspond to the spectra at 2.0 GPa and 0.5 GPa, respectively, which are superimposed by other spectra at given pressures. Vertical offset scales to the pressure measured.

In Fig. 7 we show an example of the fit to the PFY-

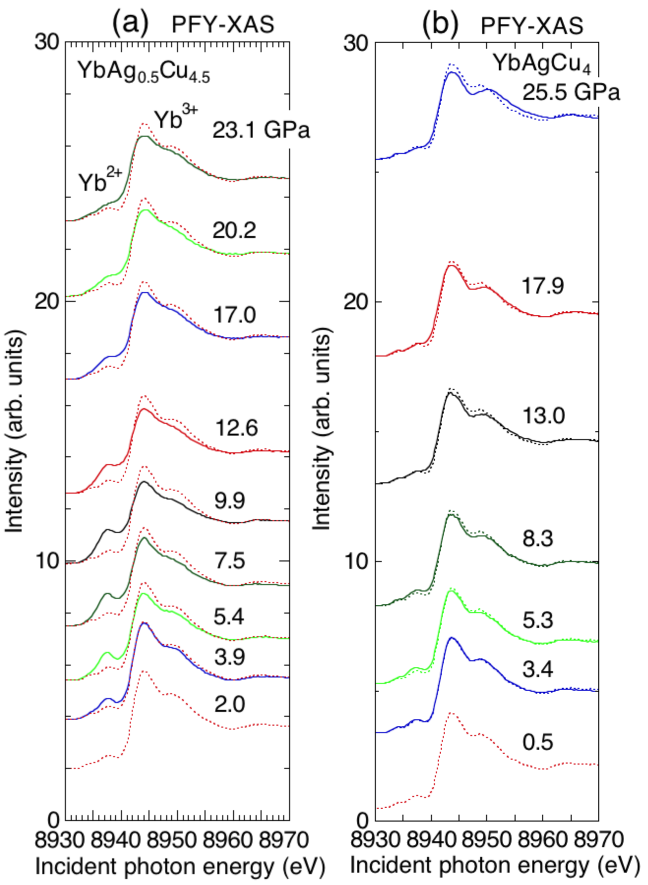

FIG. 6. (Color online). Pressure dependence of the PFY-XAS spectra of (a) $YbAg_{0.5}Cu_{4.5}$ and (b) $YbAgCu_4$ at 300 K.

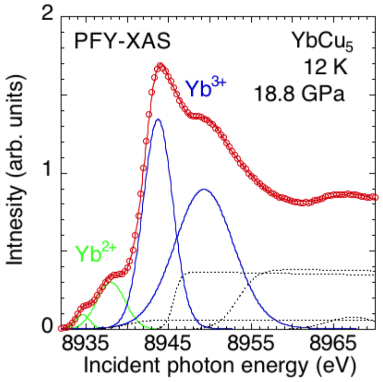

FIG. 7. (Color online). A fit example of the PFY-XAS spectrum of $YbCu_5$ at 12 K and 18.8 GPa.

XAS spectrum of $YbCu_5$ at 12 K and 18.8 GPa. Two Voigt functions are assumed for each Yb component with arctan-like backgrounds.

ELECTRONIC STRUCTURE CALCULATIONS

The calculated results are shown in Figs. 8-10. The pressure effect is considered by using the experimental lattice constants under pressure, as shown in Fig. 4, in the calculations. We have included the Yb $4f, 5d, 6s$ and the Cu $3d, 4s$ orbitals as valence states and the Yb $5s, 5p$ and Cu $3p$ orbitals as semicore states. The calculations of self-consistent loops and the density of states (DOS) are performed with a $24 \times 24 \times 24$ mesh of the reciprocal unit-cell vectors.

Figure 8 shows band dispersion weighted by the Yb-f, Cu1-d, and Cu2-d states at 0, 10, and 20 GPa. DOS at ambient pressure is shown in Fig. 9. The bands weighted by the Yb-f states show a clear splitting of the $j = 5/2$ orbitals with lower energy and $j = 7/2$ states around the Fermi level by the spin-orbit coupling, corresponding to the sharp peaks of the DOS as shown in Fig. 9. Pressure increases the width of the conduction bands and leads to the broader energy region where the f states are present through the hybridization, as clearly seen for the energy difference of the f-weighted bands at Γ and X points around the Fermi level. This leads to the reduction of the f occupation number by an order of $\sim$0.01 within the GGA approximation, but the well-developed c-f hybridization under the pressure can act to stabilize the nonmagnetic f^{14} states through the local correlation effect which is not considered in the current band calculations, as discussed below. In Fig. 10 we show the density of states near the Fermi level of Yb f, (b) Cu1 ($4c$ site) d, and Cu2 (16e site) d orbitals at 0, 10, and 20 GPa.

CRYSTAL FIELD EFFECT

Crystalline field effects (CEF) on the valence state based on the single impurity Anderson model are accounted for by [7]

$$\frac{n_f}{1-n_f} = \sum_{\Gamma} n_\Gamma V_\Gamma \rho_\Gamma \frac{1}{T_K + \Delta_\Gamma}$$
$$= \sum_{\Gamma} n_\Gamma V_\Gamma \rho_\Gamma$$
$$\times \left(\frac{1}{T_K} + \frac{1}{T_K + \Delta_1} + \frac{1}{T_K + \Delta_2} + \ldots\right), \quad (2)$$

where n_f, n_Γ, V, ρ, and Δ_i are occupation number of f states, degree of degeneracy of the Γ group, hybridization strength, density of states (DOS) at Fermi level, and crystalline electric field splittings, respectively. The crystal field strength increases with pressure, while T_K decreases. In eq. (2) the first-order expansion term changes largely with pressure, while higher-order terms do not change much, because the decrease of T_K is counterbalanced by the increase of Δ. The right-hand side of eq. (2) diverges for $T_K \to 0$. This corresponds to the Yb^{3+} state, where

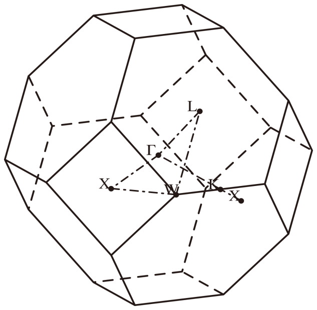

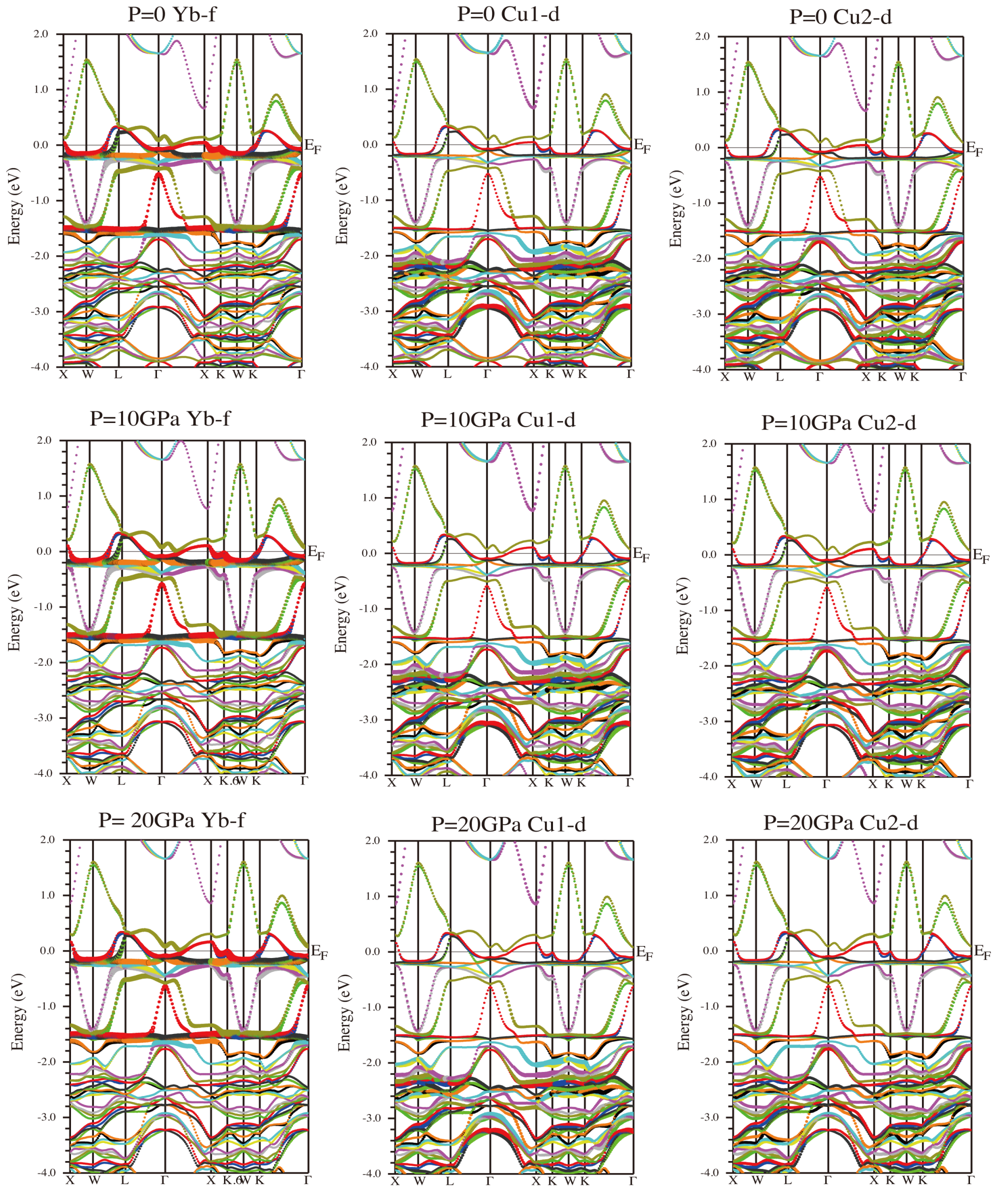

FIG. 8. (Color online). Band dispersion of cubic $YbCu_5$ weighted by the Yb f states, Cu1 d states, and Cu2 d states at 0, 10, and 20 GPa.

$n_f \approx 1$ (almost no hybridization). Therefore, in general, pressure forces the Yb valence toward the Yb^{3+} state. Assuming the validity of the Anderson model, the decrease of the Yb valence with pressure should be caused by an increase of the hybridization strength or an increase of the DOS at the Fermi level. The above band calculations suggest a possibility of the pressure-induced change in the DOS at the Fermi level. However, further theoretical and experimental studies will be necessary to understand the anomalous behavior found for cubic $YbCu_5$.

[1] Tsujii, N., He, J., Amita, F., Yoshimura, K., Kosuge, K., Michor, H., Hilscher, G., & Goto, T. Kondo-lattice formation in cubic-phase $YbCu_5$. Phys. Rev. B **56**, 8103, doi.org/10.1103/PhysRevB.56.8103 (1997).

[2] Hammersley, A. P., Svensson, S. O., Hanfland, M., Fitch, A. N., & Hausermann, D. Two-dimensional detector software: From real detector to idealised image or two-theta scan. High Pressure Research **14**, 235, doi.org/10.1080/08957959608201408 (1996).

[3] Yamaoka, H., Jarrige, I., Tsujii, N., Lin, J.-F., Hiraoka, N., Ishii, H., & Tsuei, K.-D. Temperature and pressure-induced valence transitions in

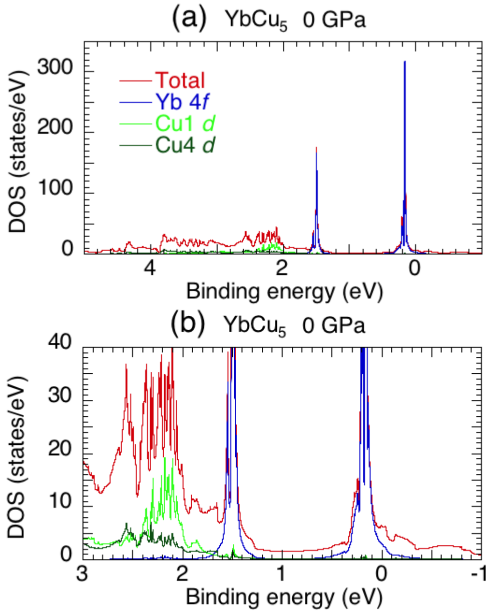

FIG. 9. (Color online). The density of states of $YbCu_5$ from Yb f, Cu1 d, and Cu2 d orbitals.

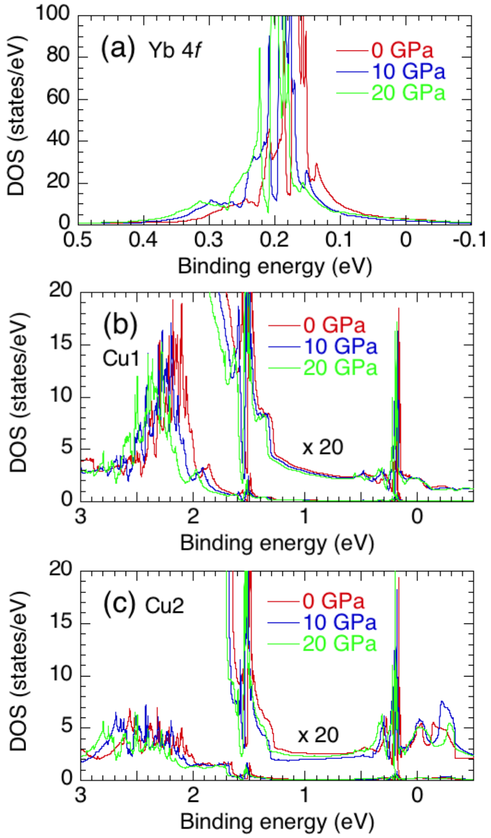

FIG. 10. (Color online). Density of states near the Fermi level of (a) Yb f, (b) Cu1 (4c site) d, and (c) Cu2 (16e site) d orbitals at 0, 10, and 20 GPa.

$YbNi_2Ge_2$ and $YbPd_2Si_2$. Phys. Rev. B **82**, 035111, doi.org/10.1103/PhysRevB.82.035111 (2010).

[4] Momma K., & Izumi, F. VESTA: a three-dimensional visualization system for electronic and structural analysis. J. Appl. Cryst. **41**, 653, doi.org/10.1107/S0021889808012016 (2008).

[5] Izumi, F. & Momma, K. Three-Dimensional Visualization in Powder Diffraction. Solid State Phenom. **130**, 15, doi.10.4028/www.scientific.net/SSP.130.15 (2007).

[6] Perdew, J. P., Burke, K., & Ernzerhof, M. Generalized gradient approximation made simple, Phys. Rev. Lett. **77**, 3865, doi.org/10.1103/PhysRevLett.77.3865 (1996).

[7] Gunnarsson O. & Schönhammer, K. Electron spectroscopies for Ce compounds in the impurity model. Phys. Rev. B **28**, 4315, doi.org/10.1103/PhysRevB.28.4315 (1983).
